# 602. Clinical Outcomes of Ertapenem in Patients with Hypoalbuminemia

**DOI:** 10.1093/ofid/ofac492.654

**Published:** 2022-12-15

**Authors:** Victoria Gavaghan, Tyler Luu, Jenna Adams, Maressa Santarossa, Fritzie S Albarillo, Megan A Rech

**Affiliations:** Advocate Lutheran General Hospital, Chicago, Illinois; Loyola University Health System, Chicago, Illinois; Loyola University Medical Center, Maywood, Illinois; Loyola University Medical Center, Maywood, Illinois; Loyola University Medical Center, Maywood, Illinois; Loyola University Health System, Chicago, Illinois

## Abstract

**Background:**

Infections caused by extended-spectrum ß-lactamase (ESBL) producing organisms pose a unique challenge for infection control. The preferred agents for treatment of infections due to ESBL-producing bacteria are carbapenems. Data from prior studies suggest that hypoalbuminemia may have a profound effect on the pharmacodynamic properties of ertapenem. Our hypothesis is that ertapenem usage in patients with hypoalbuminemia will lead to negative clinical outcomes such as infection recurrence, hospital readmission, and mortality when compared to subjects with higher albumin levels.

**Methods:**

This was a retrospective, observational, single-centered, cohort study of hospitalized patients at Loyola University Medical Center between January 2010 and August 2020. Patients were divided into 2 groups to include those who received ertapenem with serum albumin >2.5 g/dL and those who received ertapenem with serum albumin < 2.5 g/dL. The primary outcome of interest was 30-day all-cause mortality. Baseline characteristics that were collected included age, sex, nutrition status, patient comorbidities. Data regarding predictors of mortality within 24 hours of initiation of ertapenem including the Acute Physiology and Chronic Health Evaluation (APACHE) II score and the Charlson Comorbidity Index (CCI) was also collected.

Study Criteria

**Figure:**
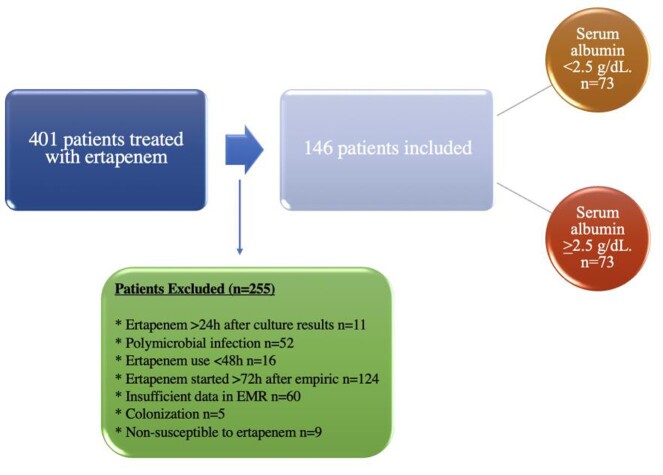

**Results:**

Of the 146 subjects that were included, 73 patients had serum albumin levels of < 2.5 g/dL during treatment with ertapenem. 30 day all-cause mortality was 19.7% for subjects with low albumin and 9.6% for subjects with normal albumin levels (p=0.09). Our study found that although not statistically significant, there is potentially a clinical significance between hypoalbuminemia and our primary endpoint, 30-day all-cause mortality, with higher rates of mortality in the low albumin group and a 9.6% between group difference. This data suggests that in subjects with hypoalbuminemia, treatment with once-daily ertapenem may lead to suboptimal outcomes regarding patient mortality.

**Figure:**
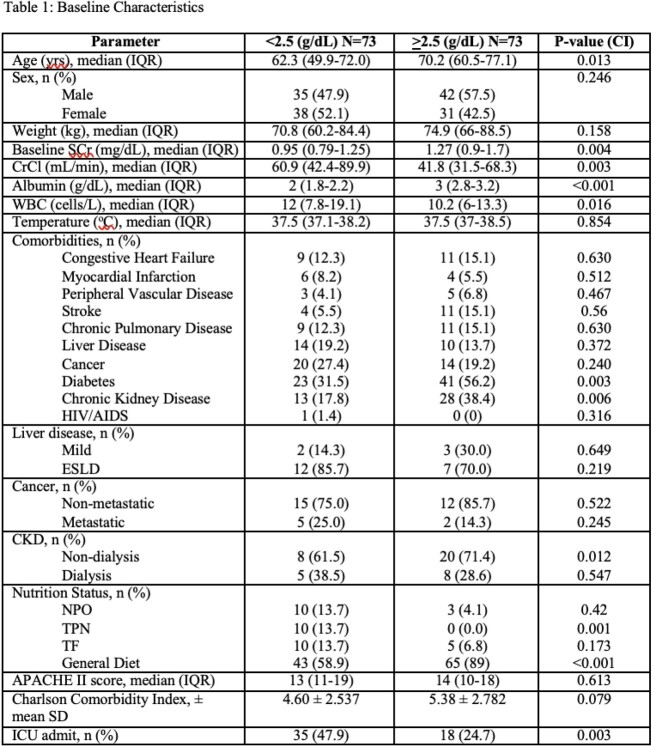

Outcome Data

**Figure:**
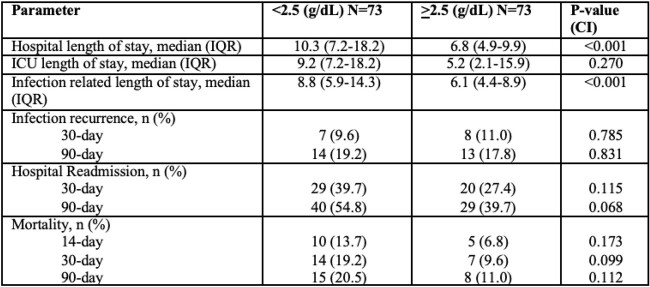

**Figure:**
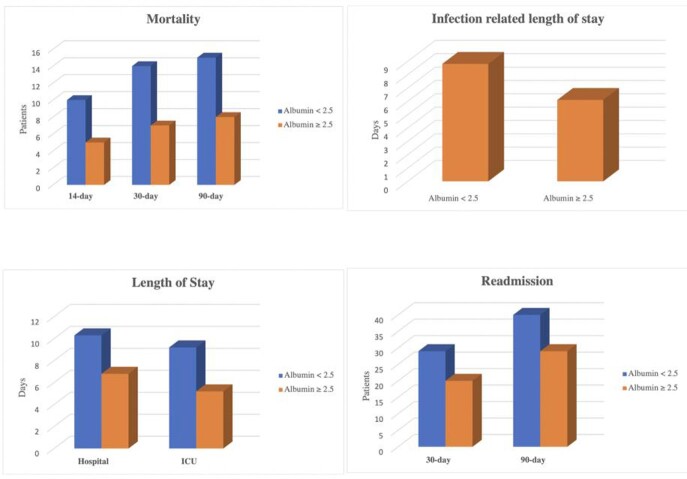

**Conclusion:**

The present study data suggests that in subjects with hypoalbuminemia, treatment with ertapenem dosed as a once-daily intravenous infusion may be associated with suboptimal clinical outcomes that may include an increased mortality, hospital readmission, and length of stay.

**Disclosures:**

**All Authors**: No reported disclosures.

